# Membranoproliferative Glomerulonephritis Due to Hepatic Hydatid Disease

**DOI:** 10.7759/cureus.92248

**Published:** 2025-09-13

**Authors:** Nabil Hamouche, Oumaima Hatimy, Mariam Chettati, Wafaa Fadili, Inass Laouad

**Affiliations:** 1 Nephrology, Mohammed VI University Hospital, Marrakesh, MAR; 2 Nephrology, Faculty of Medicine and Pharmacy of Marrakech, Cadi Ayyad University, Marrakesh, MAR

**Keywords:** case report, hydatid cyst, membranoproliferative glomerulonephritis, nephrotic syndrome, renal biopsy in nephrotic syndrome

## Abstract

Hydatid disease is a parasitic infection primarily involving the liver and is prevalent in Mediterranean and other endemic regions. Although commonly hepatic, its association with glomerular injury is extremely rare. We report the case of a 16-year-old male with a history of chronic cough and exposure to dogs who presented with generalized edema, macroscopic hematuria, and renal impairment. Laboratory evaluation confirmed nephrotic-range proteinuria, hypoalbuminemia, and impaired renal function. Renal biopsy revealed immune complex-mediated membranoproliferative glomerulonephritis (MPGN) with activation of the classical complement pathway. Imaging identified hepatic cystic lesions, and hydatid serology was positive. The patient underwent surgical excision of the cyst followed by albendazole therapy. Postoperatively, proteinuria decreased to 1.78 g/24 h by day 7 and resolved completely by day 30, with normalization of renal function and serum albumin levels. This case highlights the importance of considering hydatid disease in the differential diagnosis of unexplained nephrotic syndrome in endemic regions, as early recognition and appropriate management can lead to full recovery.

## Introduction

Membranoproliferative glomerulonephritis (MPGN) is an uncommon pattern of glomerular injury. It is characterized by excessive growth of cells within the glomeruli (mesangial proliferation) and thickening of the glomerular basement membrane, leading to a characteristic “double-contour” appearance on histology [1]. Rather than representing a single disease, MPGN is now recognized as a morphological manifestation of persistent antigenic stimulation. Secondary forms are frequently associated with chronic infections, autoimmune disorders, or monoclonal gammopathies, with viral hepatitis B and C being the leading infectious causes [2].

Hydatid disease, caused by the larval stage of *Echinococcus granulosus*, remains a significant parasitic infection in the Mediterranean basin, North Africa, and several other endemic regions. Humans acquire the infection accidentally through ingestion of parasite eggs transmitted from dogs, which serve as definitive hosts, while sheep and other herbivores act as intermediate hosts [3]. The liver is most commonly affected, followed by the lungs, while renal involvement occurs in only about 2% of cases.

Although hepatic and renal cystic localizations are well described, glomerular involvement in hydatid disease is exceedingly rare. To date, only a handful of cases of immune complex-mediated glomerulonephritis, including MPGN, have been reported in association with hydatid infection [4]. This rarity underscores the importance of recognizing unusual presentations.

Herein, we present a case of hepatic hydatid cyst (HC) associated with nephrotic syndrome secondary to MPGN. This report highlights the diagnostic and therapeutic challenges of this rare entity and emphasizes the need to consider parasitic infections in the differential diagnosis of unexplained glomerular disease in endemic regions.

## Case presentation

This case is a 16-year-old male patient with a history of chronic cough and contact with dogs. He was admitted to the nephrology department for rapidly progressive generalized edema syndrome, presenting with lower limb edema, facial puffiness, abdominal pain, chronic cough, and gross non-clotted hematuria. These symptoms occurred one month after the onset of febrile papular skin lesions. Clinical examination revealed gross hematuria, urinary dipstick showing 3+ proteinuria, and tenderness in the right hypochondrium with a feeling of heaviness in the same area.

Biological tests show normochromic normocytic anemia at 9.4 g/dL and normal leukocyte and eosinophil counts. Serum creatinine was 2.4 mg/dL (24 mg/L), with an estimated GFR of 36 mL/min/1.73m². Normal blood electrolytes. Serum protein electrophoresis was suggestive of an inflammatory syndrome. Hypoproteinemia was at 47 g/L; hypoalbuminemia at 17 g/L; 24-hour proteinuria at 4.24 g. The CRP was 40 mg/L, with normal liver function tests. Normal third and fourth components of complement (C3 and C4) levels and positive hydatid serology > 1/320 were found.

**Figure 1:**
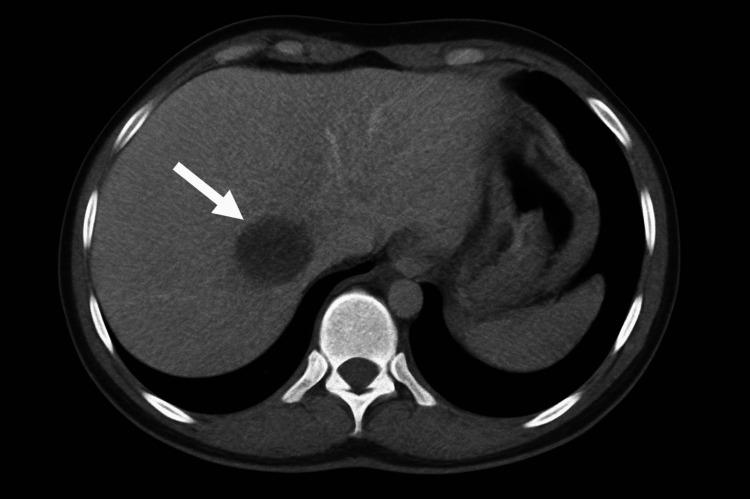
Normal-sized liver with regular contours, containing two cystic lesions in segments IV and VII, well-defined, roughly rounded, with thin walls, partially calcified, spontaneously hypodense, and non-enhancing after contrast injection, measuring 28 x 14 mm and 29 x 28 mm.

On radiological examinations, liver ultrasound showed a homogeneous liver of normal size and echostructure containing a 30 mm cystic lesion with a thickened wall. The gall bladder had a thin wall and homogeneous content. Chest X-ray showed bronchial syndrome only, and the thoraco-abdomino-pelvic CT scan (Figure 1) showed a normal-sized liver with regular contours, containing two cystic lesions in segments IV and VII, well-defined, roughly rounded, with thin walls, partially calcified, spontaneously hypodense, and non-enhancing after contrast injection, measuring 28 x 14 mm and 29 x 28 mm.

**Figure 2:**
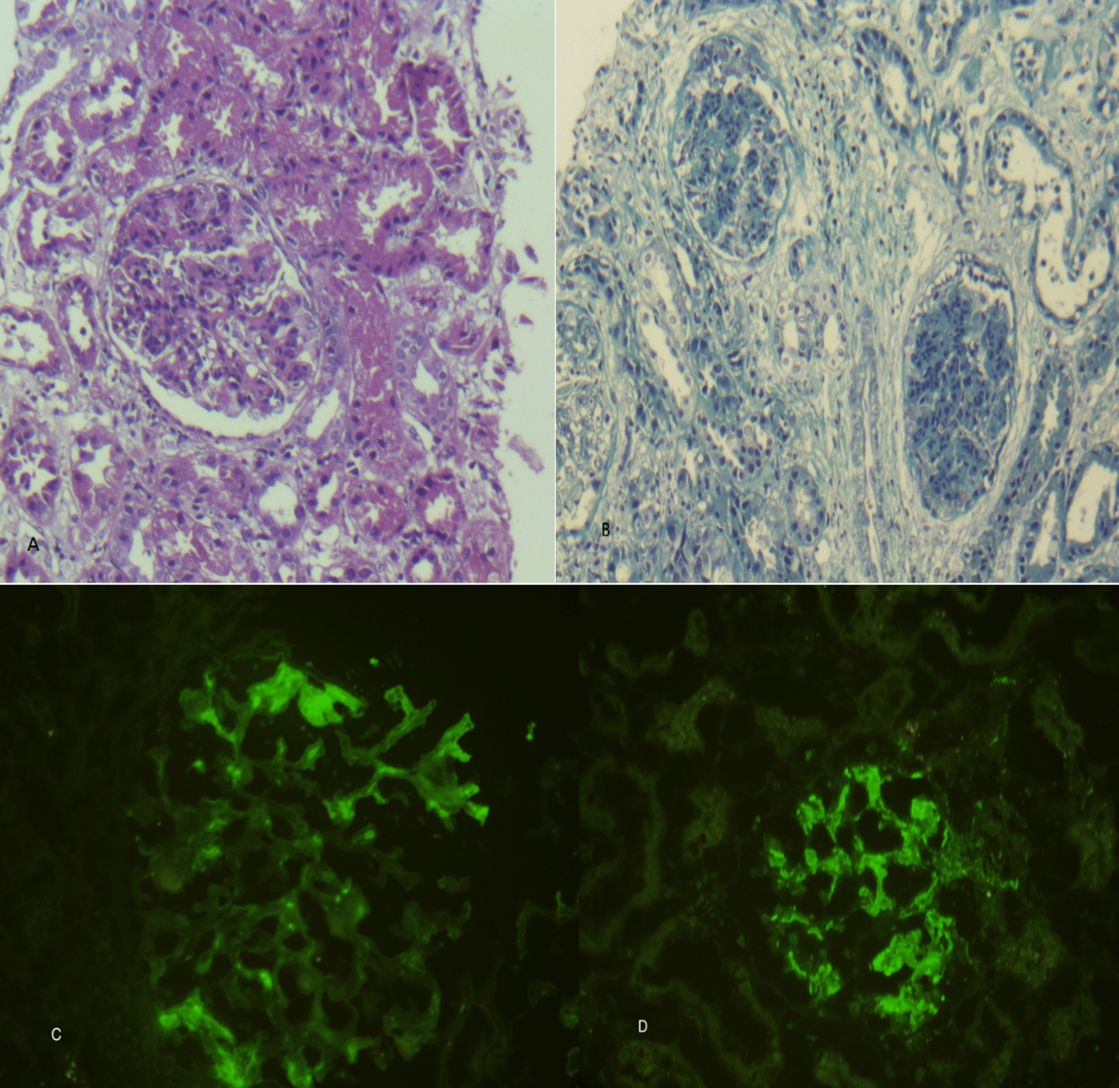
Morphological findings of the renal biopsy. (A) Hematoxylin and eosin stain (original magnification ×400) showing a glomerulus with marked hypercellularity and segmental crescent formation, indicative of active proliferative glomerulonephritis. (B) Masson’s trichrome stain (original magnification ×400) highlighting mesangial matrix expansion and areas of interstitial fibrosis. (C) Direct immunofluorescence for IgG (original magnification ×400) demonstrating granular capillary wall deposits along the glomerular basement membrane. (D) Direct immunofluorescence for C3 (original magnification ×400) showing prominent granular deposits within the glomerular capillary loops.

The renal biopsy findings are shown in Figure 2. The biopsy included 19 glomeruli, of which six had cellular crescents. There was diffuse and global involvement of all 19 glomeruli. Mild mesangial fibrosis and mesangiocapillary proliferation with capillary wall thickening and a double-contour appearance. Fairly severe interstitial nephritis with a lymphocyte-rich inflammatory infiltrate, including some plasma cells and neutrophils, and minimal interstitial fibrosis. Tubules showed red blood cell casts with minimal atrophy (~10%). Granular and subendothelial deposits showed C3, IgG, and lambda chains. 

The patient underwent surgical removal of the cyst and was started on antiparasitic treatment with albendazole. Postoperative follow-up showed a decrease in proteinuria to 1.78 g/24h by day 7 and complete resolution by day 30, along with normalization of serum albumin and serum creatinine reduced to 0.9 mg/dL (9 mg/L).

## Discussion

Hepatic HC, caused by the intrahepatic development of *Echinococcus granulosus*, is a parasitic condition that remains highly prevalent in endemic regions, including the Mediterranean basin and North Africa, where it constitutes a significant public health concern [1-3].

Although hydatid disease predominantly involves the liver and lungs, extrahepatic manifestations affecting the kidney are uncommon and typically present as renal cysts. Glomerular involvement, however, is exceptionally rare and may manifest as acute or chronic glomerulonephritis, as illustrated by our case of MPGN [2].

The life cycle of *Echinococcus* involves dogs and herbivores, primarily sheep, with humans serving as accidental intermediate hosts [3]. Following ingestion of parasite eggs, oncospheres penetrate the intestinal wall and enter the portal circulation. Most larvae are trapped in the liver or lungs, but a small proportion may bypass these filters, disseminating systemically and occasionally reaching the kidneys. Approximately 2% of cases involve renal localization, where the larvae develop into HCs [3-5].

Renal complications associated with hydatid disease are generally attributed to two main mechanisms: direct parasitic invasion and, more frequently, immunologically mediated injury. MPGN represents one such immune-mediated manifestation, likely driven by chronic antigen exposure. Leakage or rupture of HCs into the circulation allows parasite antigens to persist, facilitating immune complex formation and deposition within glomeruli. This process activates the complement cascade and promotes inflammation, ultimately resulting in the characteristic histologic features of MPGN, including mesangial proliferation and duplication of the glomerular basement membrane [1,2,5-9].

This case underscores the importance of considering parasitic infections as potential triggers of glomerular diseases, particularly in patients from endemic regions presenting with unexplained nephrotic syndrome. Several types of glomerulopathies have been reported in association with hydatid disease. While most renal manifestations involve cystic lesions, a few cases describe glomerular disease presenting with nephrotic syndrome. Reported histopathological patterns include minimal change disease, membranous nephropathy, MPGN, and other immune-mediated lesions [1,4,8-10].

Historically, Miatello et al. first documented remission of nephrotic syndrome following excision of a pulmonary HC [11]. Subsequent reports include cases of membranous nephropathy secondary to hepatic HC by Vialtel et al. and Sánchez Ibarrola et al., both resolving after cyst removal [12,13]. Covic et al. later described an elderly patient with MPGN secondary to hepatic hydatidosis who achieved complete recovery after surgical excision of the cyst [14]. Additional reports include tubulointerstitial nephritis, IgA nephropathy, and even renal amyloidosis linked to hydatid disease, with favorable outcomes after antiparasitic treatment and surgical management [9,11].

The reversibility of these lesions following eradication of hydatid infection suggests a strong causal relationship. Similarly, our patient exhibited complete remission of nephrotic syndrome and restoration of renal function after surgical cyst removal and albendazole therapy, supporting the role of early recognition and targeted treatment in achieving favorable outcomes.

## Conclusions

This case highlights the rare but clinically significant association between hepatic hydatid disease and MPGN. In patients from endemic regions presenting with unexplained nephrotic syndrome, parasitic infections should be considered in the differential diagnosis. Importantly, this case also demonstrates the reversibility of immune-mediated renal injury following timely surgical and antiparasitic treatment. Early recognition and a multidisciplinary approach are therefore essential to prevent progression to irreversible kidney damage and to achieve favorable outcomes.
